# Physical Activity and Sleep Improvements in a Group of Equestrian Therapy Volunteers: A Pilot Study

**DOI:** 10.1155/2022/5364491

**Published:** 2022-03-30

**Authors:** Inés M. Garcia-Peña, Andres García Gómez, Eloisa Guerrero-Barona, Juan M. Moreno-Manso, Sabina Barrios-Fernandez

**Affiliations:** ^1^Psychology and Anthropology Department, University of Extremadura, 06006 Badajoz, Spain; ^2^Occupational Stress, Psychopathologies and Emotional Well-Being (GRESPE) Research Group, University of Extremadura, 06006 Badajoz, Spain; ^3^Social Impact and Innovation, Health (InHEALTH) Research Group, University of Extremadura, 10003 Cáceres, Spain

## Abstract

This study aims to assess whether participation as a supportive volunteer in equestrian therapy (ET) sessions influences participants' physiological health-related parameters, including physical activity and sleep. Physical activity, measured in steps, and hours of sleep were measured in 10 subjects who participated regularly as volunteers in ET sessions using a triaxial accelerometer which continuously recorded their activity for 30 days. On the one hand, the subjects showed higher physical activity levels on days when they volunteered in ET sessions versus the days they did not. A significant difference and large effect magnitude were found. On the other hand, on the days they attended ET, they slept an average of 30 minutes more, with significant differences and a moderate effect. Thus, participation as a volunteer in ET sessions seems to have a positive influence on physical activity and sleep time, so it should be recommended as an activity to promote healthy habits.

## 1. Introduction

Equestrian therapies (ETs) are a type of animal-assisted therapy provided as a complementary activity to traditional therapies in the rehabilitation of a wide range of physical and mental disorders [1]. Usually, ET has focused on neuromuscular problems [2] and emotional and behavioural disorders [3], but these practices are becoming increasingly popular leading to research in a wide range of fields [4]. Interventions with horses require different partners in addition to the therapist; this is the reason why most equestrian centres practising therapeutic or adapted riding rely on volunteers. As horseback riding and other activities with horses are inherently dangerous, it is essential to ensure participants' safety, so these activities are usually supported by several volunteers [5, 6]. Volunteers usually include (1) the horse handler also called leader; (2) the volunteer who controls the horse from the ground, usually in a short line in front of the horse; and (3) the side handlers or assistants, who ensure the rider's safety, providing verbal instructions, rules, and positions of the different therapeutic exercises for the therapeutic activities prescribed by the specialist [7, 8].

Following an extensive bibliographical search, few studies have been found concerning volunteering in equestrian therapy (ET). In general, many volunteers take up these activities for the natural setting because they are attracted by the horses or motivated by altruism [9–11]. One of these studies indicated benefits for the volunteers' health and well-being who selflessly participated in attending therapeutic riding sessions. However, one study warns of vicarious posttraumatic stress when volunteering with users with PTSD [12]. Another aspect that should be emphasised is the volunteers' relationship with horses. Contact with animals has beneficial effects on the well-being and din personal development as well as on physical and psychological health. Because humans possess an innate emotional motivation towards other living beings, this effect is called “biophilia” [13] and [14]. Thus, riding and handling horses seem to have an added motivational value *per se* that other human activities do not have. Moreover, biophilia may increase human well-being and mental health [15]. Some studies have suggested that social interaction with other higher mammals triggers biochemical reactions, including hormones and neurotransmitters that mediate patterns of social behaviour. These interactions are based, in some way, on emotional behaviour patterns, thus producing a response in some hormones, such as cortisol, the stress hormone, and oxytocin, related to attachment and trust in relationships [16–23].

Another important aspect of ET volunteering is the physical effort required. People who volunteer to accompany users during therapy must walk with the horse for 30–60 minutes. Thus, many of those who participated in more than one session performed, at least, two hours of continuous exercise. Continuing with physical exercise, some of the benefits reported are better physical condition, meeting new people, and reducing stress levels [24]. In other cases, a significant increase in maintained calorie use after two years of participating in volunteer programmes has been reported [25]. This is relevant as previous studies have shown that, on average, women have lower mean activity levels than men [26]. As for this last idea for many years, there has been speculation about the relation between physical exercise and sleep. It is hypothesised that an increased rate of exercise may have beneficial effects on sleep in terms of duration and quality [27]. Nevertheless, it seems that there is enough scientific evidence for some medical societies to consider physical exercise as a nonpharmacological therapeutic option for sleep disorders [28].

To date, few studies have objectively studied the physical activity level of volunteers in ET sessions and, to our best knowledge, in terms of sleep quality. Therefore, this paper aims to objectively evaluate the amount of physical exercise performed by female volunteers in ET sessions and test whether the increased activity affects their sleep time.

## 2. Materials and Methods

### 2.1. Design and Variables

This work is based on an intrasubject design that records the behaviour of each individual and assesses the efficacy of the intervention compared to successive phases, as repeated measurements of the dependent variable are taken in and out of the adapted riding session [29]. The independent variable is participation in the ET sessions, and the dependent variables are a set of variables assessed using the accelerometer, including the amount of daily exercise performed, measured in steps, and the daily sleep total in minutes, the time spent in light sleep and that spent in profound sleep.

### 2.2. Participants

A convenience sample consisted of ten female volunteers collaborating in the ET sessions during the period that the research was performed. Some of the volunteers were students, and others had different labour occupations. They were from 20 to 50 years old, with a mean age of 30 years. They volunteered regularly, assisting to therapies, on average, two days a week, and on each occasion, they participated, on average, in a two-hour long sessions each day.

### 2.3. Instruments

An ADXL362 accelerometer integrated into a wristband, commercially called ‘Xiaomi Mi Band 1,' was used for the evaluation of physical activity and sleep cycles. This accelerometer is manufactured by Analog Devices Inc. and has the highest standards of reliability, following the ISO 9000 and QS 9000 standards, and the TS16949 procedures [30]. Data concerning reliability and validity point to acceptable indicators, with an accuracy of 96.6 and a precision, through the coefficient of variability, of 5.58% [31]. Furthermore, the step-counting results indicated a high correlation in steps/day between the reference, actigraphy accelerometer, and Mi Band 1 (*r* = 0.97, *p* < 0.001) [32]. Xiaomi Mi Band provided valid step count measurements in the free-living conditions. Data were extracted using the Mi Fit application version 1.5.453.

Although the device allows calories consumption estimations and distances travelled, the most reliable parameter was to measure the steps or the untransformed points of activity. In addition to the wakefulness and sleep times, the device offers 2 modes of sleep: one for light sleep and other for deep sleep, estimated by the amount of activity during sleep. However, the practice recommendations suggest that only wakefulness and sleep times should be considered since the sleep phases should be studied using polysomnography (PSG). These indicators have been kept covering a possible interest indirectly related to the quality of sleep.

### 2.4. Procedure and Ethics

Before the sessions, participants were administered the Pittsburgh Sleep Quality Index questionnaire [33] to determine whether they had any sleep disorders or irregular sleep habits that could interfere with the study, daily activity logs, and informed consent forms.

Volunteers wore the activity wristbands continuously for a month (30 days of activity and sleep periods). Volunteers, on average, participated in 2 daily ET sessions per week, so activity logs included both days with and without sessions. Each session had a duration of 60 minutes, including preparation, riding, and postriding activities. Participants were asked to follow their daily routines in terms of physical activity and to indicate whether, during the enrolment period, any exceptional incidents had occurred that would have meant abandoning the usual exercise routine, either due to excess or deficit.

Data collection was carried out at a riding centre in Salamanca (Spain), during the sessions carried out for an association aimed to provide ET for children with neurodevelopmental disorders, between 3 and 12 years of age. Three gelding horses aged 9, 11, and 15 years, trained by monitors from the Royal Spanish Equestrian Federation, were used to work with the therapists and the volunteers. These therapies were carried out in a 50m by 30m silica sand arena in the open air.

This research is part of a larger study approved by the Biosafety and Bioethics Committee of the University of Extremadura (Spain) with the approval code 77/2015.

### 2.5. Data Analysis

Given the quantitative nature of the data, a *t test* for independent samples was used to study data from the subject set and descriptive statistics as well as a nonparametric hypothesis test (Mann–Whitney *U* test) to examine each subject's case, as the data sets for the intervention phases were very short. In addition, effect size indicators (*Cohen's d*) are provided for the scores on the days volunteers attended the sessions (ET) and the scores on the days they did not attend the therapies. Values below 0.2 are considered to indicate a small effect size, 0.5 of medium magnitude, and 0.8 indicates a high effect size: small = 0.2, medium = 0.5, and large = 0.8 [34]. The free software from Psychometrica–Institut für psychologische Diagnostik *k* was used to calculate these indices [35].

## 3. Results

### 3.1. Results of the Whole Set of Volunteers

As presented in Table 1, participants covered an average of 11823 steps on the days they did not volunteer and 16469 steps when they had ET sessions, which means making 4646 additional steps. This difference is statistically significant (*p* < .000), and the magnitude of the effect is large (*d* = 0.906). The total sleep time increased an average of 30 minutes on therapy days, a statistically significant increase (*p* = 0.008) with a moderate effect (*d* = 0.363). No significant differences were found in time spent in deep or light sleep.

Table 2 presents the results of the correlation analyses between the amount of activity performed and sleep time, regardless of whether the subjects participated as volunteers in the ET sessions. The correlation between the number of steps and minutes of sleep is very low and not significant (*r* = −0.069; *p* = .259). However, a significant relationship is observed between the activity level of the subjects and the amount of deep sleep (*r* = −0.170; *p* = .005*∗∗*).

## 4. Results for Individual Participants

**Figure 1:**
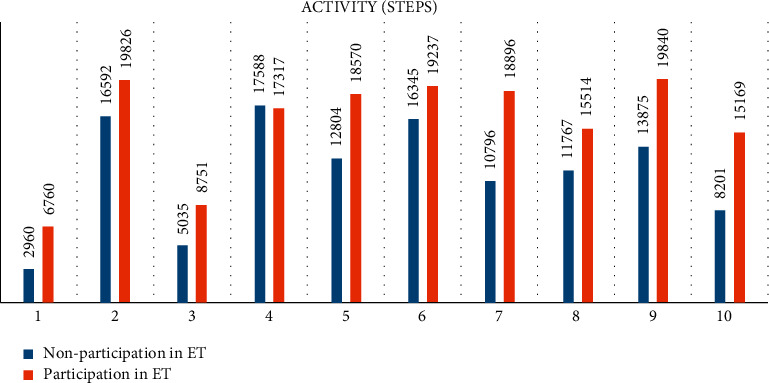
Differences in activity measured in steps before and after the intervention (equestrian therapy).

**Figure 2:**
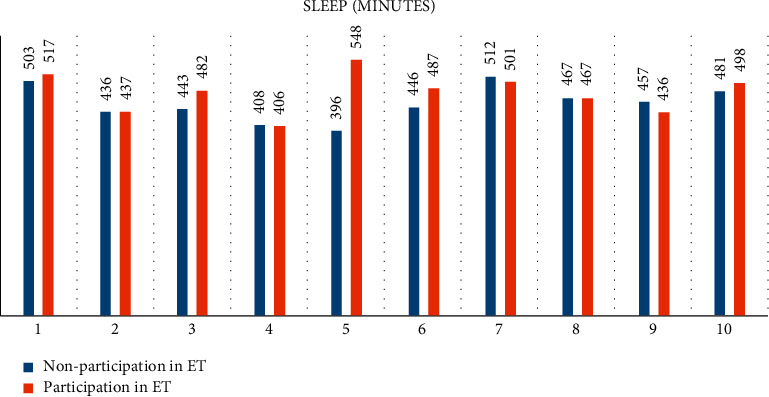
Differences in sleep measures (minutes) before and after the intervention (equestrian therapy).

While Table 3 displays descriptive and contrast statistics, Figure 1 shows the difference between physical activity and Figure 2 the difference between sleep before and after the ET sessions for each subject. As shown in Figures 1 and 2, except for participant 6, all the subjects were more active on the ET session days. One explanation is that subject 6 had a very high baseline score, which would indicate that she exercises regularly, so the therapy days did not make any additional contribution to the mean. In 7 subjects, the difference was significant and the effect size was large (*d* > 0.8).

In terms of sleeping time, 6 participants slept more on the days they attended the ET sessions. However, volunteer 8 obtained the same values in both situations; 3 of the subjects did not experience any gain, but the magnitude of the difference was smaller than that obtained by the subjects who did increase their sleeping time. Thus, 8 of the participants had more deep sleep time on ET session days, so they got a better quality of sleep or, at least, sleep time with less motor activity. Only subjects 2 and 9 had shorter deep sleep times on volunteer days. As for light sleep time, only 4 subjects increased their duration with increased motor activity, while in only 1 of them (volunteer 2), this increase was not accompanied by an increase in deep sleep. In general, when there was an increase in total sleep time, it tended to be with less motor activity.

## 5. Discussion

The results obtained indicate that participation in the ET sessions as a volunteer increases the average daily activity of the participants significantly. This increase in physical activity occurs even considering that the volunteer group consisted mainly of young women with a high baseline activity level, with an average of approximately 11823 steps walked. The baseline daily activity level of the volunteers is in the high range of activity compared to the data provided by other studies which point to average figures of 5117 steps per day in the USA, 9695 steps per day in Australia, 9650 steps in Switzerland, and 7168 steps in Japan [26]. It is important to note that a person is considered sedentary when he did not reach 5000 steps per day, and some of the participants had records close to this value at the start of the study. However, participation in the ET sessions meant that in all cases, participants exceeded 7500 steps per day on ET days [36, 37]. These results indicate that participating as a volunteer in therapeutic ET sessions involved a physical effort that helps to comply with the minimum daily activity requirements recommended by international agencies [37].

Regarding sleep, the results have shown a significant prolongation of sleeping time in the nights following volunteering. The average amount of sleep was 30 minutes longer on ET days. This study shows that participants slept an average of 7 hours and 32 minutes on ordinary days and 8 hours on ET days. One possible explanation is the beneficial physiological effect on sleep time, supported by a tendency for more restful sleep time, as lower levels of physical activity were recorded.

Previous studies, both self-reporting [38] and those using objective measurements [39], have shown that there is a moderate relation between physical activity and sleep. The effect of both acute exercise (as is the case in our study) and regular exercise on different sleep parameters has also been shown in these studies. Thus, gender, age, physical condition, the duration of the exercise, pre-exercise sleep time after exercise, and other variables may modify the effects on sleep [40]. However, in our study, we have found no significant correlation between the amount of exercise on any day and the hours of sleep on that same day. This could be due to several reasons: (1) our participants were active women, so any effect of the increased physical activity level could be attenuated since their base levels are already beyond the 7500 steps and (2) the relationship between physical activity and sleep is not so immediate as one might suppose, so moderate physical activity should be practised continuously over several weeks to influence sleep patterns [41]. Although it has not been possible to confirm a significant relationship between the physical activity level and sleeping time, our results show a moderate increase in sleep time on the days that the subjects participated as volunteers in the ET sessions. It is possible that a small effect, though not a statistically significant one, could be explained by the practice of physical activity which was the object of our study. Hence, this fact must be analysed under two specific circumstances that form part of volunteering in ET sessions: (1) the solidarity that comes with volunteering has positive effects on some physical and psychological variables, contributing to reducing stress levels and promoting physical health [42–45], as those who show concern for others and perform caring acts may be able to sleep better [46] and (2) the contact with animals can also provide a sense of well-being [47] that helps to reduce stress [18–20], favours quality of sleep [48], and improve physical and mental health [16, 17].

This work has several limitations. The study design, with no controls, and a small sample was chosen for convenience which only includes women making it not possible to generalise the results. The use of actigraphy is indicated to assess sleep time, but it does not allow to know other parameters related to sleep quality, for which polysomnography techniques would have been necessary. For this reason, measures of deep sleep and light sleep were not considered, as actigraphy only focuses on movement, and it would be required to measure breathing, brain waves, or eye movements. The use of subjective means of acquiring data may have yielded interesting information on the beneficial effects of ET on participants' sleep quality and general well-being, so future research could include a qualitative approach. Therefore, these data should be taken as preliminary results, and further studies are needed to confirm our findings, using larger samples including both sexes and research designs that allow generalization.

## 6. Conclusions

This work provides preliminary evidence that volunteering in ET sessions could have a positive influence on participants' areas of the physical activity level and sleep and could therefore be a well-being promoting activity.

## Figures and Tables

**Table 1:** Descriptive and contrast statistics for all the subjects.

Variable	Nonparticipation in equestrian therapies	Participation in equestrian therapies	*t*	*p* ^*a*^	*d* ^*b*^
Activity (steps)	11823.78 ± 6369.52	16469.99 ± 4587.54	6.71	.000 ^*∗∗*^	0.906
Total sleep	452.34 ± 86.78	483.30 ± 80.97	2.68	.008 ^*∗∗*^	0.363
Deep sleep	196.04 ± 87.35	209.37 ± 86.46	1.13	.258	0.153
Light sleep	260.64 ± 102.43	276.79 ± 87.62	1.20	.228	0.163

^a^ *p* (level of statistical significance:  ^*∗*^*p* < 0.05,  ^*∗∗*^*p* < 0.01). ^b^Cohen's *d*: small effect 0.2–0.49, medium effect 0.5–0.79, and large effect >0.8.

**Table 2:** Pearson's correlation coefficients and levels of significance between physical activity and sleep.

		Activity (steps)	Total sleep	Deep sleep	Light sleep
Activity (steps)	*r* ^*a*^	1	−.069	−.170	.094
	(*p*^*b*^)		(.259)	(.005^∗∗^)	(.122)
Total sleep	*r*		1	.373	.536
	(p)			(.000^∗∗^)	(.000^∗∗^)
Deep sleep	*r*			1	−.540
	(p)				(.000^∗∗^)
Light sleep	*r*				1
	(p)				

^a^Pearson's correlation coefficients. ^*b*^*p* (level of statistical significance: ^∗^*p* < 0.05, ^∗∗^*p* < 0.01).

**Table 3:** Descriptive and contrast statistics.

Subject Age (years)	Variable	Nonparticipation in Equestrian Therapies	Participation in Equestrian Therapies	*Mann–Whitney U test*	*Z*	*p* ^*a*^	*d* ^*b*^
122	Activity	2960 ± 1297.04	6760 ± 1786.2	6.0	−3.760	.000^∗∗^	2.020
	Total sleep	503.57 ± 50.85	516.75 ± 59.62	67.00	−0.478	.633	0.180
	Deep sleep	271.42 ± 59.84	306.12 ± 70.34	56.50	−1.036	.300	0.400
	Light sleep	239.57 ± 62.83	210.37 ± 45.15	55.00	−1.110	.265	0.439

220	Activity	16592.1 ± 5220.8	19826.5 ± 1346.5	18	−1.095	.273	0.458
	Total sleep	436.21 ± 60.32	437.33 ± 44.29	28	−0.048	.962	0.019
	Deep sleep	235.26 ± 55.05	220 ± 31.19	20	−0.813	.416	0.336
	Light sleep	199.73 ± 63.45	217.33 ± 41.78	23.5	−0.478	.632	0.196

350	Activity	5034.88 ± 1325.85	8751 ± 626.16	0.0	−3.162	.002^∗∗^	1.450
	Total sleep	443.5 ± 28.77	482.25 ± 56.06	26	−1.446	.148	0.557
	Deep sleep	280 ± 50.33	310.25 ± 34.63	32	−1.051	.293	0.398
	Light sleep	162.83 ± 39.63	172 ± 27.36	37.5	−0.690	.490	0.258

448	Activity	17587.76 ± 6446.9	17317.66 ± 4549	55	−0.467	.641	0.177
	Total sleep	408.68 ± 115.68	406.66 ± 125.46	60	−0.336	.737	0.127
	Deep sleep	250.36 ± 98.81	256.16 ± 98.11	66	0.000	1.000	0.000
	Light sleep	171.95 ± 68.85	170 ± 98.89	62.5	−0.196	.845	0.074
524	Activity	12804.16 ± 4450.4	18570.0 ± 4629.81	42	−2.517	.012^∗∗^	0.957
	Total sleep	395.88 ± 84.17	548.62 ± 84.72	17	−3.487	.000^∗∗^	1.527
	Deep sleep	186.96 ± 48.80	248.75 ± 54.63	37.50	−2.626	.009^∗∗^	1.027
	Light sleep	208.52 ± 59.00	299.87 ± 97.73	42.0	−2.437	.015^∗∗^	0.936

644	Activity	16345 ± 6739.34	19237.66 ± 360.69	18.0	−1.095	.273	0.388
	Total sleep	445.8 ± 107.12	487.66 ± 46.52	19.0	-1.004	.315	0.355
	Deep sleep	70.15 ± 27.11	84.31 ± 56.58	30	0.000	1.000	0.000
	Light sleep	383.55 ± 92.77	403.43 ± 90.86	26	−0.365	.715	0.127

723	Activity	10796.52 ± 3391.1	18896.54 ± 4020.1	10.00	−4.151	.000^∗∗^	2.323
	Total sleep	512.50 ± 100.13	501.08 ± 92.41	100.50	−0.319	.750	0.116
	Deep sleep	210.6 ± 70.38	215.5 ± 77.43	100.50	−0.319	.750	0.116
	Light sleep	301.82 ± 56.84	285.63 ± 69.66	89.50	−0.786	.432	0.290

823	Activity	11766.87 ± 3681.5	15514.50 ± 3537.2	34.00	−2.424	.015^∗∗^	1.080
	Total sleep	467.46 ± 96.94	467.00 ± 88.44	71.50	−0.194	.846	0.077
	Deep sleep	158.33 ± 31.98	193.30 ± 80.08	59.50	−0.860	.390	0.349
	Light sleep	309.13 ± 71.24	283.70 ± 49.68	56.00	−1.054	.292	0.431

921	Activity	13875.16 ± 6079.2	19839.75 ± 1657.5	32.00	−2.785	.005^∗∗^	1.101
	Total sleep	457.28 ± 79.94	436.62 ± 57.76	78.50	−0.903	.366	0.311
	Deep sleep	139.37 ± 49.08	127.12 ± 23.89	85.50	−0.457	.648	0.151
	Light sleep	322.91 ± 66.06	319.00 ± 66	93.5	−0.273	.785	0.092

1024	Activity	8201.64 ± 3115.52	15169.36 ± 3393.3	11.00	−3.613	.000^∗∗^	2.092
	Total sleep	481.42 ± 45.08	498.27 ± 36.76	52.50	−1.342	.180	0.557
	Deep sleep	130.78 ± 70.03	157.63 ± 43.76	44.00	−1.807	.071	0.775
	Light sleep	372.64 ± 112.44	340.63 ± 40.71	70.00	−0.383	.702	0.153

^a^ *p* (level of statistical significance: ^∗^*p* < 0.05, ^∗∗^*p* < 0.01). ^b^Cohen's *d*: small effect 0.2–0.49, medium effect 0.5–0.79, and large effect >0.8.

## Data Availability

The datasets used to support the findings of this study are available from the corresponding author upon request.

## References

[1] Farias-Tomaszewski S., Jenkins S. R., Keller J. (2001). An evaluation of therapeutic horseback riding programs for adults with physical impairments. *Therapeutic Recreation Journal*.

[2] Snider L., Korner-Bitensky N., Kammann C., Warner S., Saleh M. (2007). Horseback riding as therapy for children with cerebral palsy: is there evidence of its effectiveness?. *Physical & Occupational Therapy in Pediatrics*.

[3] Frewin K., Gardiner B. (2005). New age or old sage? A review of equine-assisted psychotherapy. *The Australian Journal of Counselling Psychology*.

[4] Enders-Slegers M.-J., Hediger K., Beetz A., Jegatheesan B., Turner D. (2019). Animal-assisted interventions with an international perspective: Trends, research, and practices. *Handbook on animal-assisted therapy: Foundations and guidelines for animal-assisted interventions*.

[5] Naidoo R., Nqwena Z., Reimers L. (2014). Acute heart rate variability responses to a therapeutic horseback riding session in children with autism spectrum disorders: a pilot study. *Scientific and Educational Journal of Therapeutic Riding*.

[6] Peter C. C. S., Shuler N. J., Jones S. H. (2021). Comparing training methods to improve volunteer skills during therapeutic horseback riding: a randomized control trial. *Journal of Applied Behavior Analysis*.

[7] Professional Association of Therapeutic Horsemanship International (2011). *Riding Instructor On-Site Workshop Manual*.

[8] Bickman C., Casey J. (2012). *Special Olympics Equestrian Coaching Guide*.

[9] Scott N., Evans J. W. (2005). *Special Needs, Special Horses: A Guide to the Benefits of Therapeutic Riding*.

[10] Salay J. (2010). *A Narrative Inquiry of Volunteer Experiences at a Midwestern Equestrian Facility for Individuals with Disabilities*.

[11] Buswell D., Leriou F. (2007). Perceived benefits of students’ service-learning experiences with hippotherapy. *Faculty Publications*.

[12] Ben-Porat A., Gil L., Brafman D., Zriker A., Levy D. (2021). Secondary traumatic stress and posttraumatic growth among volunteers at a therapeutic riding center: the role of personal and environmental factors. *Current Psychology*.

[13] Cutt H., Giles-Corti B., Knuiman M., Burke V. (2007). Dog ownership, health and physical activity: a critical review of the literature. *Health & Place*.

[14] Wilson E. O. (1984). *Biophilia EEUU*.

[15] Gullone E. (2000). The biophilia hypothesis and life in the 21st century: increasing mental health or increasing pathology?. *Journal of Happiness Studies*.

[16] Odendaal J. S. J. (2000). Animal-assisted therapy - magic or medicine?. *Journal of Psychosomatic Research*.

[17] Johnson R. A., Meadows R. L. (2002). Older latinos, pets, and health. *Western Journal of Nursing Research*.

[18] Viau R., Arsenault-Lapierre G., Fecteau S., Champagne N., Walker C.-D., Lupien S. (2010). Effect of service dogs on salivary cortisol secretion in autistic children. *Psychoneuroendocrinology*.

[19] Cho S.-H., Kim J.-W., Kim S.-R., Cho B.-J. (2015). Effects of horseback riding exercise therapy on hormone levels in elderly persons. *Journal of Physical Therapy Science*.

[20] Tabares C., Vicente F., Sánchez S., Aparicio A., Alejo S., Cubero J. (2012). Quantification of hormonal changes by effects of hippotherapy in the autistic population. *Neurochemical Journal*.

[21] Beetz A., Julius H., Kotrschal K., Uvnäs-Moberg K. (2011). Basic neurobiological and psychological mechanisms underlying therapeutic effects of equine assisted activities (EAA/T). *HHRF research grant application*.

[22] Johnson R. A., Johnson P. J., Megarani D. V. (2017). Horses working in therapeutic riding programs: cortisol, ACTH, glucose, and behavior stress indicators. *Journal of Equine Veterinary Science*.

[23] Melco A. L., Goldman L., Fine A. H., Peralta J. M. (2020). Investigation of physiological and behavioral responses in dogs participating in animal-assisted therapy with children diagnosed with attention-deficit hyperactivity disorder. *Journal of Applied Animal Welfare Science*.

[24] O’Brien L., Townsend M., Ebden M. (2010). ‘Doing something positive’: volunteers’ experiences of the well-being benefits derived from practical conservation activities in nature. *Voluntas: International Journal of Voluntary and Nonprofit Organizations*.

[25] Tan E. J., Rebok G. W., Yu Q. (2009). The long-term relationship between high-intensity volunteering and physical activity in older African American women. *Journals of Gerontology Series B: Psychological Sciences and Social Sciences*.

[26] Bassett D. R., Wyatt H. R., Thompson H., Peters J. C., Hill J. O. (2010). Pedometer-measured physical activity and health behaviors in United States adults. *Medicine & Science in Sports & Exercise*.

[27] Banno M., Harada Y., Taniguchi M. (2018). Exercise can improve sleep quality: a systematic review and meta-analysis. *PeerJ*.

[28] Santos R. V. T., Tufik S., De Mello M. T. (2007). Exercise, sleep and cytokines: is there a relation?. *Sleep Medicine Reviews*.

[29] Barlow D. H., Nock M. K., Hersen M. (2008). *Single Case Experimental Designs: Strategies for Studying Behavior Change*.

[30] *Analog Devices, Inc, Reliability Handbook UG-311. Norwood, MA 02062-9106*.

[31] El-Amrawy F., Nounou M. I. (2015). Are currently available wearable devices for activity tracking and heart rate monitoring accurate, precise, and medically beneficial?. *Healthcare Informatics Research*.

[32] Tam M. K., Cheung S. Y. (2019). Validation of consumer wearable activity tracker as step measurement in free-living conditions. *Finnish Journal of eHealth and eWelfare*.

[33] Carralero García P., Hoyos Miranda F. R., Sandoval D., López García M. (2013). Calidad del sueño según el Pittsburgh Sleep Quality Index en una muestra de pacientes recibiendo cuidados paliativos. *Medicina Paliativa*.

[34] Cohen J. (1988). *Statistical Power Analysis for the Behavioral Sciences*.

[35] Lenhard A., Lenhard W. (2016). *Psychometrica: Calculation of Effect Sizes*.

[36] Tudor-Locke C., Bassett D. R. (2004). How many steps/day are enough?. *Sports Medicine*.

[37] del Pozo Cruz B., Gallardo-Gomez D., Ding D. (2021). How many steps a day to reduce the risk of all-cause mortality? A dose-response meta-analysis. *Journal of Internal Medicine*.

[38] Youngstedt S. D., Kline C. E. (2006). Epidemiology of exercise and sleep. *Sleep and Biological Rhythms*.

[39] Loprinzi P. D., Cardinal B. J. (2011). Association between objectively-measured physical activity and sleep, NHANES 2005–2006. *Mental Health and Physical Activity*.

[40] Kredlow M. A., Capozzoli M. C., Hearon B. A., Calkins A. W., Otto M. W. (2015). The effects of physical activity on sleep: a meta-analytic review. *Journal of Behavioral Medicine*.

[41] Baron K. G., Reid K. J., Zee P. C (2013). Exercise to improve sleep in insomnia: exploration of the bidirectional effects. *Journal of Clinical Sleep Medicine*.

[42] Jenkinson C. E., Dickens A. P., Jones K. (2013). Is volunteering a public health intervention? A systematic review and meta-analysis of the health and survival of volunteers. *BMC Public Health*.

[43] Yeung J. W. K., Zhang Z., Kim T. Y. (2017). Volunteering and health benefits in general adults: cumulative effects and forms. *BMC Public Health*.

[44] Wu A. M. S., Tang C. S. K., Yan E. C. W. (2005). Post-retirement voluntary work and psychological functioning among older Chinese in Hong Kong. *Journal of Cross-Cultural Gerontology*.

[45] Whillans A. V., Dunn E. W., Sandstrom G. M., Dickerson S. S., Madden K. M. (2016). Is spending money on others good for your heart?. *Health Psychology*.

[46] Leitner D. (2009). *The Relation between Well-Being, Sleep, Benevolence, and Personality*.

[47] Friedmann E., Son H., Tsai C.-C. (2010). The animal/human bond. *Handbook on Animal-Assisted Therapy*.

[48] Headey B., Na F., Zheng R. (2008). Pet dogs benefit owners’ health: a ‘natural experiment’ in China. *Social Indicators Research*.

